# Laparoscopic Management of a Migrated Intrauterine Device Causing a Partial-Thickness Rectal Injury Diagnosed Following Contraceptive Failure: A Case Report

**DOI:** 10.7759/cureus.101538

**Published:** 2026-01-14

**Authors:** Nehemia Kassa, Efeson Malore, Yosef Alemayehu A Shalemo, Kaleb Tsega, Yabets Abode

**Affiliations:** 1 Surgery, Myungsung Christian Medical Center, Addis Ababa, ETH; 2 General and Colorectal Surgery, Myungsung Christian Medical Center, Addis Ababa, ETH; 3 General Surgery, Myungsung Christian Medical Center, Addis Ababa, ETH; 4 Global Health, Duke University, Durham, USA; 5 Internal Medicine and Surgery, Myungsung Christian Medical Center, Addis Ababa, ETH

**Keywords:** case report, contraceptive complications, iud migration, laparoscopic surgery, rectal perforation, uterine perforation

## Abstract

Intrauterine devices (IUDs) are widely used long-acting reversible contraceptives due to their efficacy and safety. Although generally well tolerated, uterine perforation with subsequent migration into adjacent pelvic or abdominal organs is a rare but potentially serious complication. Rectal involvement is exceptionally uncommon and often clinically silent. We report the case of a 32-year-old woman who presented with complete abortion of an unintended pregnancy 18 months after IUD insertion. On physical examination, the IUD strings could not be visualized, and transvaginal ultrasound failed to locate the device within the uterine cavity. An abdominopelvic X-ray identified the IUD within the pelvic cavity. Diagnostic laparoscopy revealed the device embedded in the anterior rectal wall, resulting in a partial-thickness rectal wall injury. The IUD was successfully removed, and the rectal defect was repaired laparoscopically. The patient’s postoperative course was uneventful, and she remained asymptomatic at follow-up. This case contributes to the limited literature on rectal wall injury secondary to IUD migration and highlights how such cases may remain undetected without a high index of suspicion and appropriate imaging. Early recognition and timely minimally invasive surgical management are essential to ensure optimal patient outcomes and to prevent serious complications, including abscess formation, fistula development, and bowel injury. This report underscores the importance of considering IUD migration and possible pelvic organ injury in patients presenting with missing strings or unintended pregnancy.

## Introduction

Intrauterine devices (IUDs) are among the most widely used forms of reversible contraception worldwide, valued for their high efficacy, long-acting nature, and convenience. Over 150 million women globally rely on IUDs, making them a cornerstone of family planning programs [1]. Their widespread acceptance is further supported by their safety and suitability for a broad range of women, including those who are postpartum or breastfeeding, with effectiveness exceeding 99%.

Although generally safe, IUDs can be associated with rare but potentially serious complications. Uterine perforation, occurring in approximately 1-2 per 1,000 insertions, is the most significant immediate mechanical complication [2]. Following perforation, the device may remain partially within the uterus or migrate beyond the uterine cavity, potentially involving adjacent organs such as the bladder, omentum, and, rarely, the GI tract [2,3]. Migration usually occurs at the time of insertion but may also present weeks to months later due to gradual pressure necrosis of the uterine wall caused by the IUD, uterine contractions, or localized inflammation [3]. Risk factors include recent childbirth or abortion, lactation, uterine anomalies, improper insertion technique, and placement by inexperienced providers [2,3].

Gastrointestinal involvement occurs in roughly 15% of IUD migration cases, with the most commonly affected segments being the sigmoid colon and small intestine [4]. Rectal involvement is particularly uncommon and represents a small proportion of reported gastrointestinal IUD migration cases, most of which are described as isolated case reports [5,6]. Clinical presentation is highly variable, ranging from asymptomatic cases to complications such as abdominal or pelvic pain, rectal bleeding, fistula formation, or bowel obstruction, which can delay diagnosis [5,6]. Timely diagnosis therefore relies on a structured evaluation when warning signs appear. The diagnostic pathway typically begins with ultrasound when IUD strings are missing or an unintended pregnancy occurs, followed by plain radiography if the device is not intrauterine, and cross-sectional imaging (CT or MRI) when complications are suspected or when precise localization is needed [7,8].

We present a case of an IUD that migrated and caused a partial-thickness rectal injury, which was successfully managed laparoscopically. This report highlights the importance of early recognition of both subtle and overt signs of IUD migration, outlines a practical diagnostic approach, and demonstrates the role of minimally invasive surgery in managing such cases and preventing severe complications.

## Case presentation

A 32-year-old gravida 2, para 2 woman presented to the ED with a 12-hour history of vaginal bleeding. She had two prior term pregnancies and no history of miscarriage or abortion before the current presentation. She was 18 months postpartum following an uncomplicated spontaneous vaginal delivery. The patient had exclusively breastfed for the first six months postpartum; her menstrual cycles resumed thereafter and remained regular until the current presentation. Her last menstrual period was three weeks prior to presentation. Her medical and surgical histories were unremarkable, and she was not taking any long-term medications.

The patient reported that a copper-containing T-shaped IUD of an unspecified model had been inserted one month after her previous delivery as a contraceptive measure. The insertion was performed by an experienced obstetrician in an outpatient setting and was uncomplicated, with no immediate post-procedural pain or abnormal bleeding. Ultrasound guidance was not used at the time of insertion, as it was not routinely employed. Following insertion, she experienced no pelvic pain, abnormal vaginal bleeding, gastrointestinal symptoms, systemic complaints, or history suggestive of spontaneous IUD expulsion. She attended a routine follow-up visit one week after IUD insertion, during which pelvic examination confirmed appropriate positioning of the device with visible retrieval strings and no abnormalities noted. She remained asymptomatic and in good health during outpatient follow-up until her current presentation.

**Figure 1:**
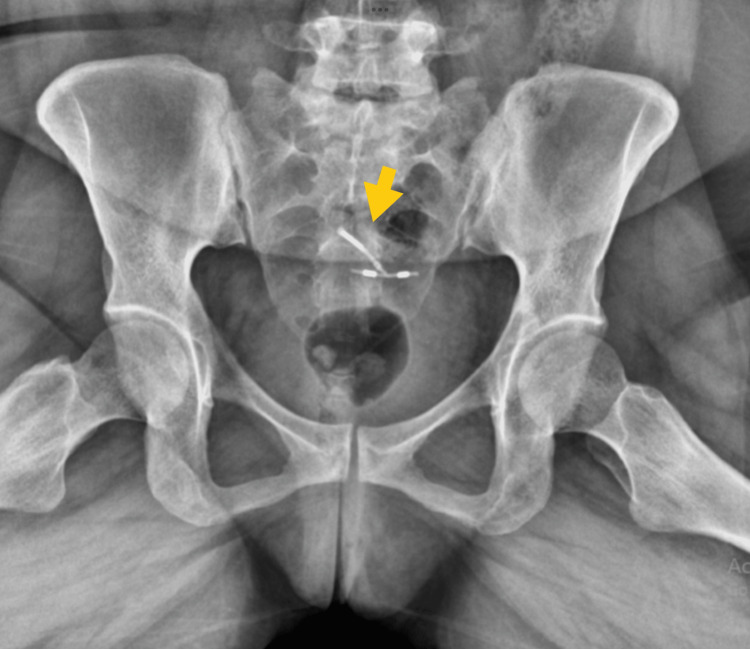
Pelvic X-ray demonstrating an anteroposterior (AP) view of the IUD in the pelvic cavity (yellow arrow). IUD: Intrauterine device.

On evaluation, pelvic examination revealed active vaginal bleeding and absence of visible IUD strings. A qualitative urine β-human chorionic gonadotropin (β-hCG) test was positive. Transvaginal USG demonstrated findings consistent with a complete abortion of an intrauterine pregnancy, including an empty uterine cavity with a thin endometrial lining measuring 10 mm and no retained products of conception. Both adnexa appeared normal, with no adnexal masses or free fluid identified, thereby excluding ectopic pregnancy. However, the IUD was not visualized within the uterine cavity. After stabilization, the patient underwent further evaluation to determine the cause of contraceptive failure. A plain abdominal radiograph was obtained, which demonstrated a T-shaped radiopaque structure within the pelvis (Figure 1), consistent with an extrauterine IUD.

The patient was subsequently transferred to the surgical service for further management. Following multidisciplinary evaluation, diagnostic laparoscopy was planned for localization and removal of the migrated IUD. The device was clearly localized on plain radiography, the patient was hemodynamically stable, and there were no clinical findings suggestive of complicated bowel involvement. In addition, advanced cross-sectional imaging was not readily available. For these reasons, further imaging with CT or MRI was deferred.

**Figure 2:**
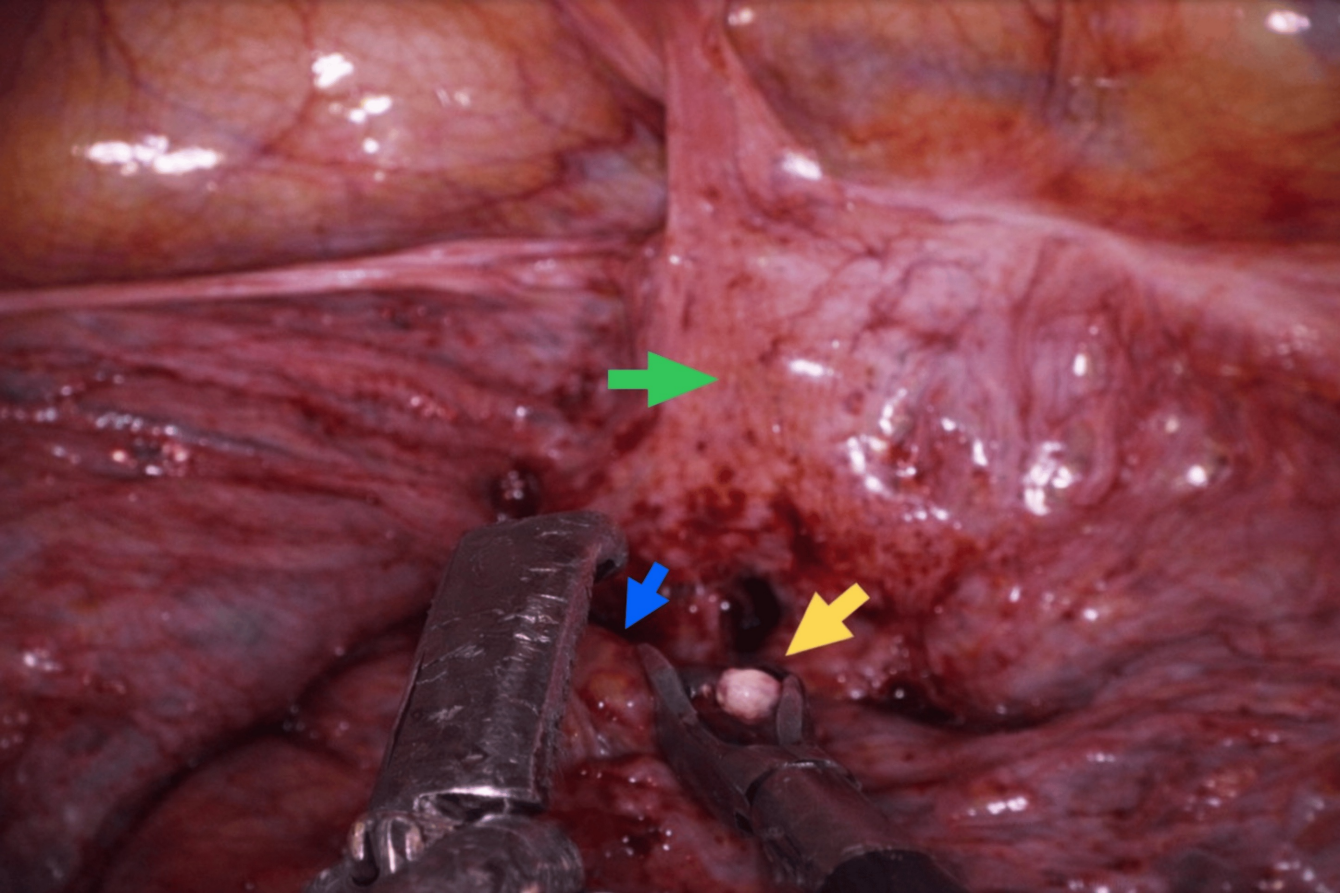
IUD (yellow arrow) localized and encased by dense adhesions between the rectum (blue arrow) and uterus (green arrow), with one arm perforating the rectal serosa. IUD: Intrauterine device.

Laparoscopic access was achieved using three ports placed at the umbilicus, right lower quadrant, and left lower quadrant. Intraoperative inspection revealed an intact uterus with no visible evidence of perforation. Dense localized adhesions were identified between the posterior uterine wall and the rectum. Following careful adhesiolysis, the IUD was visualized embedded within the anterior rectal wall, with one horizontal arm perforating the rectal serosa (Figure 2). There was no evidence of fecal contamination, pelvic abscess formation, or generalized peritonitis.

**Figure 3:**
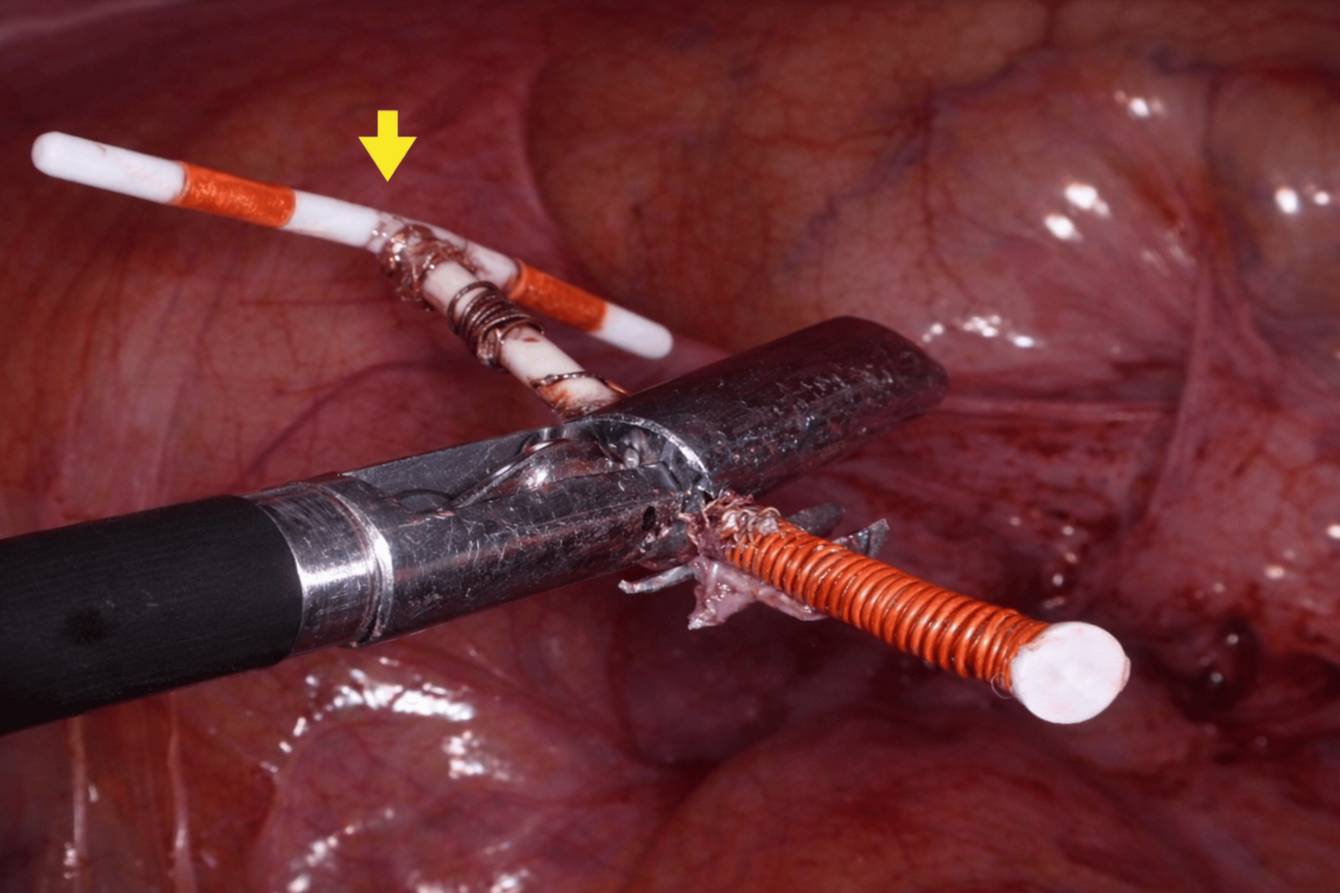
IUD removed laparoscopically without complications (yellow arrow). IUD: Intrauterine device.

**Figure 4:**
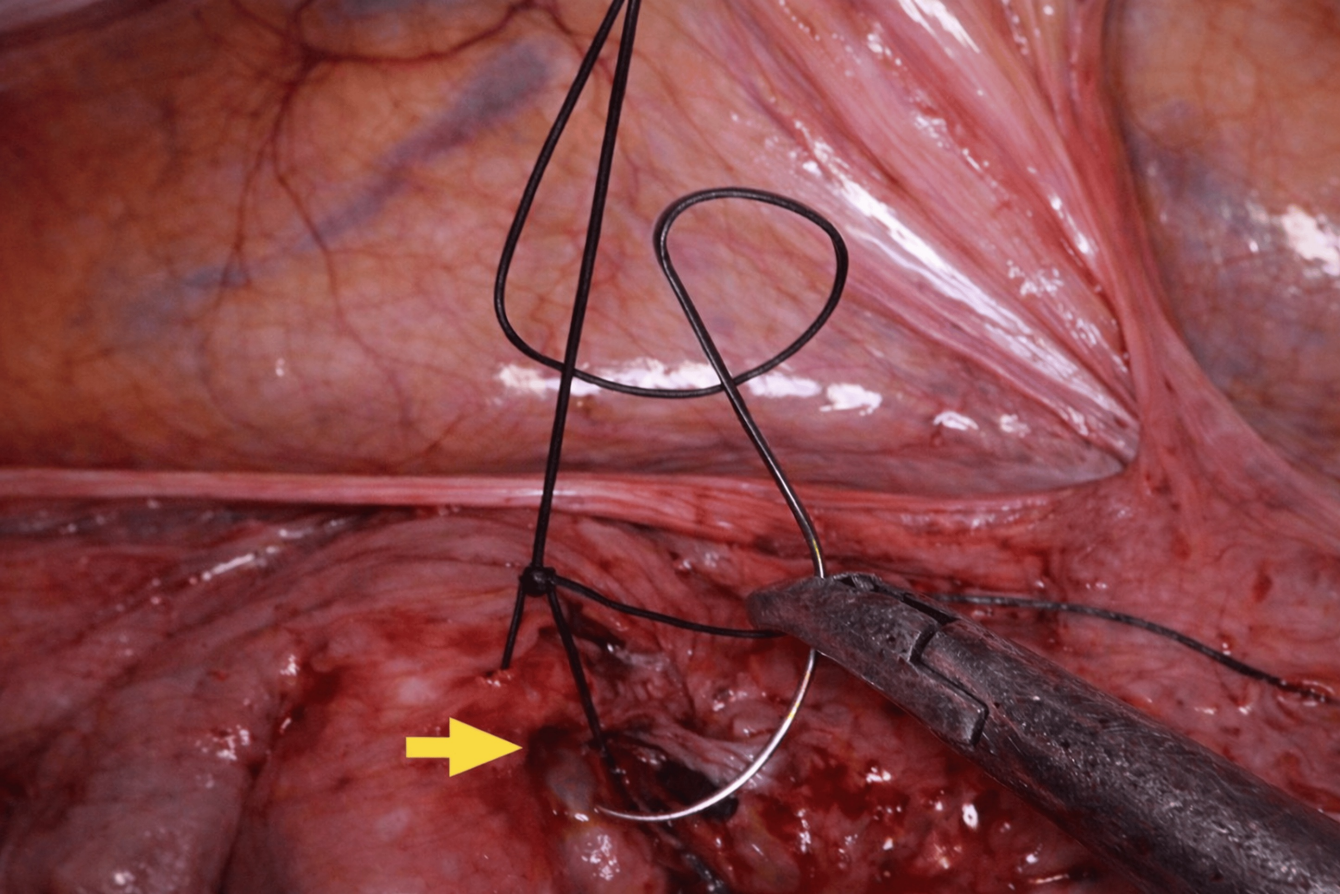
Rectal defect (yellow arrow) closed with absorbable sutures.

Intraoperative proctoscopy confirmed the absence of intraluminal rectal involvement. The IUD was carefully dissected and removed laparoscopically (Figure 3). The rectal defect was classified as a partial-thickness injury, involving the serosa and superficial muscularis without mucosal penetration. Full-thickness perforation was excluded intraoperatively through direct laparoscopic visualization, which confirmed no transmural defect, and proctoscopy, which demonstrated no intraluminal communication. The defect was repaired using interrupted 2-0 Vicryl sutures in a seromuscular fashion (Figure 4), with no omental patch reinforcement required. An air-leak test was then performed intraoperatively to assess the integrity of the repair by insufflating the rectum with air while the pelvis was filled with saline, and was negative. The total operative time was approximately 75 minutes, with an estimated blood loss of less than 50 mL. No intraoperative complications occurred, conversion to laparotomy was not required, and no intra-abdominal drain was placed.

The patient’s postoperative course was uneventful. She received intravenous prophylactic antibiotics (ceftriaxone and metronidazole) for three days, followed by oral Augmentin and metronidazole for five days. She was kept nil per os (NPO) for 12 hours, then advanced to a clear liquid diet on postoperative day 1, followed by a soft diet as tolerated. Normal bowel function returned on the first postoperative day. The patient remained clinically stable throughout her hospital stay and was discharged on postoperative day 2. At six-week evaluation, she remained asymptomatic with no gastrointestinal or gynecologic complaints. No additional complications were observed during subsequent outpatient follow-up up to three months.

## Discussion

Uterine perforation is a rare but serious complication of IUD use. Data from the European Active Surveillance Study on Intrauterine Devices (EURAS-IUD) report a risk of approximately 1.3 per 1,000 insertions for copper IUDs and 1.2 per 1,000 insertions for levonorgestrel IUDs, with higher risk in early postpartum women and those who are lactating [3]. Additional risk factors include uterine anatomical variations and insertion by less experienced providers [2,3]. While risk is broadly similar between copper and levonorgestrel devices, slight differences may reflect study populations or provider experience rather than the device itself [3,7]. In this case, postpartum placement and ongoing lactation were present, likely contributing to uterine perforation and migration.

Perforations may be partial or complete, with complete perforation allowing migration of the device into the peritoneal cavity and involvement of adjacent organs [2,7]. Extrauterine migration of IUDs can occur at various sites. The bladder is a recognized site of migration; in such cases, the device may act as a nidus for stone formation or cause urinary symptoms, often requiring cystoscopic or surgical removal [8]. Migration into the omentum or general peritoneal cavity may be asymptomatic or cause vague abdominal pain [2,4]. GI tract involvement, while rare, carries risks of bowel perforation, obstruction, fistula, or abscess formation, emphasizing the need for early diagnosis and surgical management [4-6].

Rectal involvement following IUD migration is exceptionally rare [5,6]. Clinical presentation can be subtle; patients may remain asymptomatic for long periods, and migration is often discovered incidentally during evaluation for missing IUD strings, unintended pregnancy, or unexplained pelvic pain [7,9]. Our patient presented with an unintended pregnancy and absent IUD strings but reported no GI symptoms, underscoring the silent nature of some migrations. When the rectum is involved, potential complications include rectal bleeding, fistula formation, bowel obstruction, or abscess [5,6], highlighting the importance of timely diagnosis.

Accurate imaging is crucial for the diagnosis and localization of migrated IUDs. Transvaginal or transabdominal ultrasound is typically the first-line modality, but extrauterine devices may be missed, necessitating further imaging with plain abdominal X-ray, CT, or MRI for precise localization [7,8]. CT or MRI is particularly indicated when there is suspicion of complications such as abscess, fistula, or full-thickness bowel injury; when the exact location of a migrated IUD is unclear; or in patients with severe abdominal or pelvic symptoms. However, in settings where resources are limited, including many low- and middle-income countries, plain radiography may be sufficient when the device is clearly visualized and the patient is clinically stable, as in our case. This approach allows safe and timely surgical planning while avoiding unnecessary radiation exposure, additional costs, and delays in treatment [5,8]. In our case, the IUD was clearly visualized on X-ray, the patient was stable, and there were no signs of complex complications, supporting the decision to proceed directly to diagnostic laparoscopy, which is a feasible approach in selected scenarios.

Surgical removal of a migrated IUD is recommended even in asymptomatic patients to prevent potentially serious complications. Laparoscopy is generally preferred because of its minimally invasive nature, superior visualization, and favorable postoperative recovery profile [5,7]. Alternative management strategies depend on the location of the device and the extent of organ involvement. Colonoscopic or transanal retrieval has been described in cases where the IUD is completely intraluminal and not associated with serosal injury or adhesions [5,9], while combined laparoscopic-endoscopic approaches may be useful in selected cases with partial bowel wall involvement [6,8]. Open surgical removal is typically reserved for complicated presentations, including extensive adhesions, bowel obstruction, fistula formation, abscess, or generalized peritonitis [2,5]. In our case, laparoscopy was optimal as it allowed for safe dissection of dense adhesions, assessment and repair of the rectal injury, and confirmation of repair integrity, fulfilling both diagnostic and therapeutic roles.

Prevention of IUD perforation relies on careful insertion technique. Insertion should ideally be performed at least 6 weeks postpartum, with ultrasound confirmation considered in higher-risk settings [3,7]. Patients should receive counseling on warning signs, including persistent pelvic pain, abnormal bleeding, or missing strings, and should be instructed to seek prompt evaluation in cases of contraceptive failure or inability to visualize IUD strings. Regular follow-up, particularly in patients with known risk factors such as postpartum placement or lactation, remains essential to ensure proper device position and early detection of complications [9].

## Conclusions

This case illustrates that IUD migration may present with unintended pregnancy and absent retrieval strings. Although rectal involvement is rare, migrated IUDs can result in clinically significant bowel injury even in the absence of gastrointestinal symptoms. Careful and timely evaluation using appropriate imaging is important to localize the device and assess for associated organ injury. When selected appropriately, minimally invasive laparoscopic management can allow safe removal of the migrated device, accurate assessment of injury extent, and effective repair while minimizing surgical morbidity. This report supports consideration of IUD migration in patients with contraceptive failure or missing strings and demonstrates that laparoscopic intervention is a feasible and effective management option in selected cases.
